# Interdisciplinary management of patients with special healthcare needs undergoing dental treatment in a tertiary care hospital setting in Germany: a retrospective study

**DOI:** 10.1007/s40368-025-01023-8

**Published:** 2025-03-28

**Authors:** N. Schulz-Weidner, M. Hofmann, C. Uebereck, N. Krämer, M. A. Schlenz, V. Becker, F. Edinger, D. Leicht, M. F. Müller, T. S. Zajonz

**Affiliations:** 1 Dental Clinic – Department of Paediatric Dentistry, Justus Liebig University, Schlangenzahl 14, 35392 Giessen, Germany; 2 Department of Prosthodontics, Christian Albrecht University of Kiel, University Hospital Schleswig-Holstein, Campus Kiel, Kiel, Germany; 3 Department of Anesthesiology, Operative Intensive Care Medicine and Pain Therapy, University Hospital of Giessen and Marburg, Campus Giessen, Rudolf-Buchheim-Str. 7, 35392 Giessen, Germany

**Keywords:** General anaesthesia, Retrospective study, Paediatric dentistry, Interdisciplinary study, Special healthcare needs, Dental treatment

## Abstract

**Purpose:**

The aim of this retrospective study was to analyse the dental and medical parameters, including peri- and postoperative management and complications, of patients with special healthcare needs receiving dental treatment in a tertiary care hospital setting.

**Methods:**

A total of 154 patients (mean age 7.8 ± 4.1 years) who received dental treatment under general anaesthesia or analgosedation at the Department of Paediatric Dentistry of the XXX University in XXXXXXX between 2021 and 2023 were divided into the following diseases: metabolic disease, nervous system disorder, congenital heart disease, tumour disease, gastroenterological disease, genetic syndrome, pulmonology disease and coagulopathy. Caries experience (dmf-t/DMF-T), type of anaesthesia and pre- and perioperative parameters were recorded.

**Results:**

Regardless of disease, all children showed higher caries experience in the primary dentition compared to permanent dentition (mean ± standard deviation; 6.44 ± 4.85/2.01 ± 3.87). Most of the children suffered from genetic syndrome, followed by congenital heart and metabolic disease. Dental treatment was mostly performed under general anaesthesia. 92.2% of those patients were intubated orotracheally and 66.9% received antiemetics. The complication rate was lower than 3%.

**Conclusions:**

Data show that special healthcare needs patients regardless of kind of disease are highly affected by caries and require dental treatment. Most dental restorations were performed under general anaesthesia. Regardless of disease and type of anaesthesia, the complication rate was low, which underlines the high clinical relevance of adequate dental care under general anaesthesia for this vulnerable patient group.

## Introduction

Dental caries still remains a global public health burden (Mota-Veloso et al. 2016). Although a decrease in the prevalence of dental caries has been observed in recent decades, there is a significant group of children with very high levels of dental caries and subsequent treatment needs requirering extensive dental treatment under general anaesthesia (GA) for various reasons (Khanh et al. 2015). The need for timely caries treatment is undeniable. Without treatment, tooth decay can cause pain and infection (Mota-Veloso et al. 2016). In addition, the disease can affect the general health of children (Hayden et al. 2013).

However, regular dental treatment with the use of local anaesthesia is challenging for children with pre-existing cofactors, known as special healthcare needs (SHCN) (Sari et al. 2014). At the same time, children with SHCN have an even higher caries experience and a severe need of dental treatment than children without general disease due to the negative impact of the general disease on oral hygiene and healthy nutrition (Schulz-Weidner et al. 2022).

The increasing number of SHCN patients and the need to care for them poses challenge for the entire dental team. Many organisations have published guidelines regarding the appropriate use of sedation and GA in dentistry amongst SHCN patients and healthy children without general disease (Coté and Wilson 2019; Rahman et al. 2022). Appropriate dental care under GA is required to improve the effectiveness and safety of treatment and to establish best clinical practise. Accordingly, for patients with SHCN, the interdisciplinary management between dentist, dental nurse and anaesthetist is a prerequisite for dental treatment under GA with minimal side effects (Wang et al. 2012). These limiting conditions often require health care interventions through specialised services (Academy of Pediatric Dentistry. Council on Clinical Affairs 2012). Safe Anaesthesia for Every Tot (SAFETOTS) has derived ten rights as essential for paediatric anaesthesia (Safetots.org) like SmartTots underlining the importance of focussing safe and comfortable medical care for children undergoing anaesthesia and sedation (Ramsay and Rappaport 2011). Moreover, the European Society for Paediatric Anaesthesiology (ESPA) has established guidelines on the training, organisation and practise of paediatric anaesthesia and acting as a centre of expertise for paediatric anaesthesiology.

Oral rehabilitation can improve not only oral health-related quality of life of the patients, including their social and emotional well-being, as well as their growth and even their blood chemistry parameters, such as ferritin and vitamin D (Ferrazzano et al. 2020). Due to the possible dental complications and associated risks of anaesthesia, particularly, these SHCN patients are usually rehabilitated in an inpatient setting in tertiary care dental clinics. In our Department of Paediatric Dentistry in XXXX (XXXX (XXX), Germany) these children with SHCN are regularly treated as inpatients under GA or analgosedation (AS) in cooperation with the Paediatric Center (Medical Centre of Paediatrics, XXX,XXXX) and the Clinic for Anaesthesia (Pedaitric Heart Center, Department of Anesthesiology, operative Intensive Care and Pain Therapy, XXXX,XXXX).

Dental treatment in this clinical setting offers the advantages of trained staff, interdisciplinary reliability, constant monitoring and adequate management of complications by specialised and highly trained treatment units. In this way, possible complications and risks can be avoided as far as possible (Dziedzic 2017). Moreover, standardised protocols improve quality of anaesthesia for patients.

Nasotracheal intubation (NTI) represents an established airway management technique in dental treatment and surgery, though it presents distinct challenges compared to oral intubation. These include increased technical difficulty, longer procedure duration, and potential complications such as nasal trauma, bleeding, necrosis, retropharyngeal perforation, sinusitis and more pronounced post extubation atelectasis (Black et al. 1990; Holzapfel 2003; Jagannathan et al. 2015). Current evidence suggests no significant differences between nasal and oral routes regarding nosocomial pneumonia, bacteremia, and otitis (Holzapfel 2003). The technical challenges of NTI primarily involve passage through the nasal cavity, navigation in the oropharynx, and alignment with the glottic opening (Khoo 2020). Whilst technique refinements have improved success rates, the paediatric population presents unique considerations. Notable gaps exist in the literature regarding paediatric-specific guidelines for tube sizing and insertion depth (Kim and Jeon 2024). Recent evidence from dental surgery applications has demonstrated significant disadvantages of NTI compared to oral intubation (Ciccozzi et al. 2024). A pilot study revealed longer mean intubation times (6.3 vs 2.1 min, *p* < 0.01), increased provider requirements, and higher trauma rates (69% vs 0%, *p* < 0.01) with NTI. Age distribution analysis showed that oral intubation was predominantly used in younger patients (mean age 5.9 years) compared to NTI (mean age 7.3 years, *p* = 0.01) (Bowman et al. 2018). In close cooperation with the dentistry department and optimal continuous monitoring of airway protection (fixation, leakage, etc.), safe implementation of oral intubation as standard airway management was possible, even in complex high-risk patients, without compromising dental or anaesthesiological management.

With regard to the fact that anaesthetic agents include potentially emetic substances (Kovac 2021), recommendations for postoperative nausea and vomiting (PONV) must be taken into account. PONV can lead to paediatric morbidity, delays in discharge and unplanned inpatient stays (Kovac 2021). Various prophylactic approaches and rescue treatment medications, such as 5-HT3-receptor-inhibitors, droperidol and promethazine, are recommended depending on the age and risk group (Gan et al. 2020). In consequence, the possibilities of anaesthesia have improved significantly in recent years, so that GA has become more tolerable. This allows easier dental treatment of children, including those with diseases, who in themselves have a more limited compliance than healthy children. Up to today, there is a lack of data for this special group of children receiving dental treatment under anaesthesia. Therefore, the present study aimed to analyse the dental and medical parameters as well as peri- and postoperative complications of a study collective with SHCN patients receiving dental treatment under GA or AS in a hospital setting. Furthermore, it should be studied to what extent factors such as caries experience effects the type of anaesthesia.

The main null hypotheses for this study were formulated as follows:

1. there is no association between caries experience (dmf-t/DMF-T) and disease;
2. there is no association between severity of caries experience and management of anaesthesia;
3. there is no difference in terms of peri- and postoperative complications between the different patient groups.

## Methods

### Subjects and setting

The present study was designed as a retrospective cohort study, which included data from all patients with general diseases of the Department of Paediatric Dentistry (XXXX, Germany) who received a dental treatment under anaesthesia between 2021 and 2023.

All data were collected in compliance with the ethical principles of the Declaration of Helsinki and the survey was approved by the Ethics Committee of the Faculty of Medicine of the XXXXXXX University XXXXXXXXX (Ref. no. 104/23). The standard written informed consent for dental treatment under anaesthesia was obtained from caregivers before treatment. Exclusion criteria were incompleteness of medical records and missing written informed consent.

### Data collection

All existing files of the Department of Paediatric Dentistry were searched for the main target parameter of the present study (dental treatment under anaesthesia). All children treated under anaesthesia were registered in a database. Data were extracted from the digital patient records of the patient data management system (Meona, Mesalvo, Freiburg, Germany; NarkoData, IMESO-IT GmbH, Giessen, Germany).

The following parameters were considered: age, gender, type of general disease of the patient, American Society of Anesthesiologists (ASA)-Classification, type of anaesthesia, type of intubation and type of dental treatment being performed. In the case of multiple diagnoses, the first-named diagnosis was included according to ICD-10 after reviewing the doctor’s letter. The dmf-t/ DMF-T value for the primary/permanent teeth was recorded for caries experience. A tooth was considered as decayed (d/D) if a carious lesion (without consideration of initial carious lesions) was found, as missing (m/M) if the reason for loosening the tooth was caries, and filled (f/F), if there was a restoration (Pieper and Blumenstein 1993). In addition, the type of inpatient follow-up care and any complications and side effects, such as allergic reactions, nausea, vomiting, need for reintubation or resuscitation, were documented.

### Statistical analysis

The statistical data analysis was carried out using the software programme SPSS (version 29.0, IBM Corporation, Armonk, New York, NY, USA).

The results of the descriptive statistics were presented as absolute and relative frequencies. The inductive statistics included the investigation of correlations or differences between the type of intubation and the occurrence of nausea and vomiting as well as the use of an antiemetic, between the severity of the dental findings (dmf-t/ DMF-T value) and the type of GA and type of intubation used. The Kruskal–Wallis test with Bonferroni correction and the Mann Whitney test were used for this purpose.

A significance level of 5% was defined for the entire statistical analysis.

### Ethics approval and consent to participate

The local ethics committee of the XXXX approved this study (Ref. no. 104/23). All methods were carried out in accordance with relevant guidelines and regulations. Data collection was anonymous. No images are presented in this article. The need for written informed consent was waived by the Justus Liebig University Giessen ethics committee due to the retrospective nature of the study.

## Results

One hundred fifty four patients (81 males, 73 females) aged between one to 23 (mean: 7.8 ± 4.1) years of age were enrolled. Table 1 shows the distribution of the different types of general diseases within the study cohort. Combination diagnoses occurred in 52 cases. The most common of these was complex neurological disease combined with congenital heart disease, followed by genetic syndrome and congenital heart disease. A confirmed genetic syndrome, such as Trisomie 21 (*n* = 8), Angelman Syndrome (*n* = 3) and Koolen-de Vries Syndrome (*n* = 3) was present in 18.18% of the patients. 14.29% of the patients suffered from a congenital heart disease, such as Fallot’s Tetralogy (*n* = 7) or hypoplastic left heart syndrome (*n* = 5) just as many of the patients suffered from a metabolic disease, such as hypothyroidism or diabetes. 11.69% showed a pulmonological disease, for example bronchial asthma, hypoplastic lungs or surfactant deficiency, 10.39% a disorder of the nervous system, for example epilepsy or cerebral palsy. Gastroenterological diseases, tumour diseases and coagulopathies were present in less than 7% of the cases (Table 1).

**Table 1:** Absolute and relative frequencies of types of general diseases within the study cohort

	Absolute frequency [*n*]	Relative frequency [%]
Total number of patients	154	100.0
Type of general disease		
Metabolic disease	22	14.29
Nervous system disorder	16	10.39
Congenital heart disease	22	14.29
Gastroenterological disease	8	5.19
Complex neurological disease	21	13.64
Genetic syndrome	28	18.18
Tumour disease	10	6.49
Pulmonological disease	18	11.69
Coagulopathy	9	5.84

### American Society of Anesthesiologists (ASA)-classification

The majority of patients were classified to ASA 3, followed by ASA 2. Only 4.50% were allocated to ASA 1 (Table 2), this was never the case for metabolic disease, congenital heart disease, genetic syndromes and tumour disease.

**Table 2:** Absolute frequencies of types of American Society of Anesthesiologists (ASA)-Classification within the study cohort

	ASA 1 absolute frequency [*n*]	ASA 2 absolute frequency [*n*]	ASA 3 absolute frequency [*n*]
Type of general disease			
Metabolic disease	*n* = 7	5	17
Nervous system disorder		4	11
Congenital heart disease		7	15
Gastroenterological disease		4	3
Complex neurological disease		13	5
Genetic syndrome		11	17
Tumour disease		4	6
Pulmonological disease		6	11
Coagulopathy		4	4
Total	7	58	89

### dmf-t/DMF-T value

Before dental treatment under anaesthesia, the average dmf-t value was 6.44 (mean) ± 4.85 (standard deviation) with an average of 6.15 ± 4.78 decayed, 0.16 ± 0.78 missing and 0.13 ± 0.66 filled primary teeth. For the permanent dentition, an average DMF-T value of 2.01 ± 3.87 with an average of 1.98 ± 3.84 decayed, no missing (0.00) and no filled teeth (0.07 ± 0.55) could be observed. Table 3 shows the distribution of the mean dmf-t/DMF-T value for every general disease showing highest value for the primary dentition in patients with tumour disease followed by pulmonological disease whereas in permanent dentition highest value was shown for genetic syndrome patients.

**Table 3:** Mean dmf-t/DMF-T value of types of general diseases within the study cohort

		dmf-t (Mean ± SD)	DMF-T [Mean] and standard deviation (SD)
Metabolic disease	*n* = 22	6.11 ± 4.76	1.33 ± 1.90
Nervous system disorder	*n* = 16	6.28 ± 4.52	2.12 ± 2.32
Congenital heart disease	*n* = 22	6.10 ± 4.70	2.23 ± 5.10
Gastroenterological disease	*n* = 8	6.13 ± 2.80	2.13 ± 5.62
Complex neurological disease	*n* = 21	6.22 ± 4.99	2.62 ± 3.46
Genetic syndrome	*n* = 28	6.30 ± 5.57	3.28 ± 5.39
Tumour disease	*n* = 10	9.50 ± 3.17	0.70 ± 1.34
Pulmonological disease	*n* = 18	6.61 ± 5.37	1.44 ± 2.01
Coagulopathy	*n* = 9	6.56 ± 3.54	0.00 ± 0.00

SD: standard deviation

**Fig. 1:**
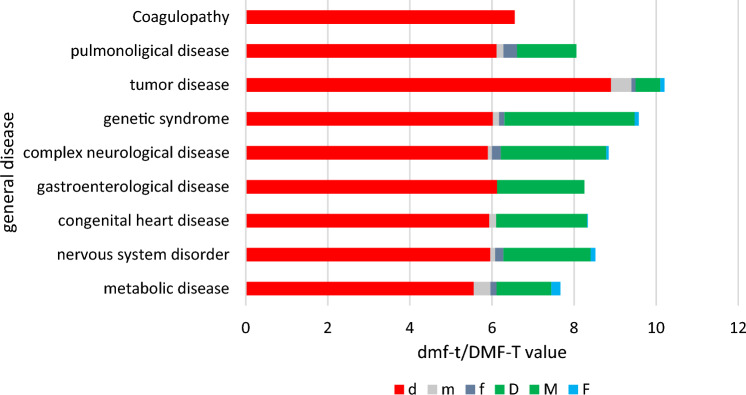
Distribution of the dmf-t/ DMF-T values and the single values before dental treatment. d/D = number of decayed teeth. m/M = number of missing teeth. f/F = number of filled teeth. dmf-t/DMF-T = sum of the decayed, missing and filled teeth

Figure 1 shows the distribution of the number of destroyed, missing or filled teeth for every disease in primary and permanent teeth revealing that the proportion of the dmf-t value consisted mainly of teeth affected by caries. Fillings and missing teeth due to extractions played a subordinate role (Fig. 1).

**Fig. 2:**
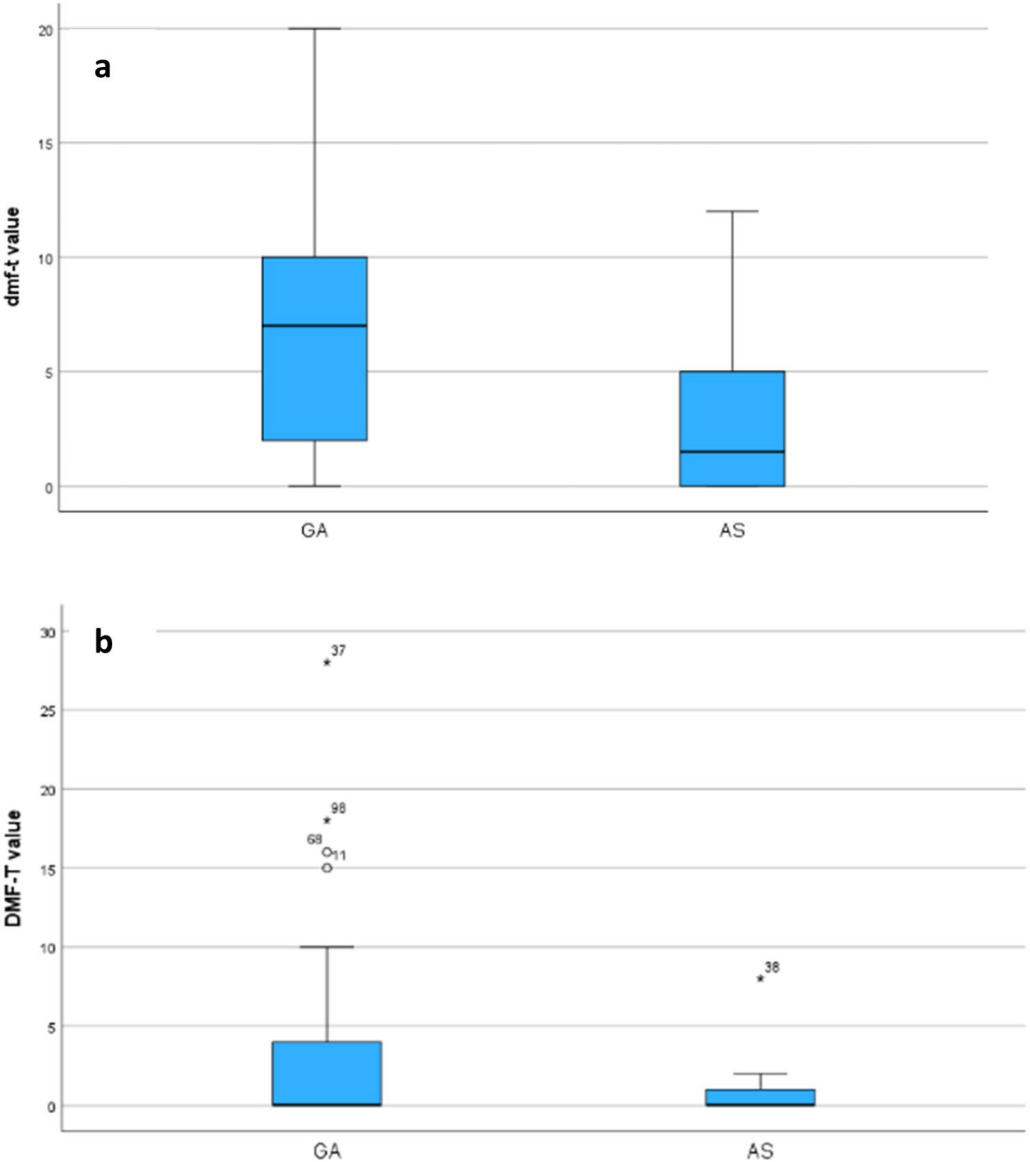
Distribution of **a** dmf-t (primary dentition) and **b** DMF-T (permanent dentition) values of the patients according to the type of anaesthesia (*p* < 0.003; **Kruskal–Wallis** test). Central lines: medians. Boxes: interquartile Range (IQR). Whiskers: 10% quantile/90% quantile. Circles: outliers 1 (= values below/above whiskers but within 1.5 × IQR). Stars: outliers 2 (= values below/above 1.5 × IQR)

For the primary teeth, patients that were treated under AS showed significantly lower dmf-t values (median dmft-t values: 1.5 [AS], 7.0 [GA]) than patients receiving GA (Fig. 2a; *p* < 0.003, *Kruskal–Wallis* test). No significant differences were observed between the types of anaesthesia with respect to permanent teeth DMF-T values (median DMF-T values: 0.0 for both groups; Fig. 2b; *p* > 0.05, *Kruskal–Wallis* test). Figure 2 shows the distribution of the dmf-t/DMF-T values according to the type of anaesthesia the patients received.

The most common dental treatment (37.1% of the cases) was solely tooth extraction, followed by restorative therapy including fillings and stainless steel crowns (33.8% of the cases), and by the combination of restorative therapy and tooth extraction (27.2%). Osteotomies and combinations with other head and neck surgical procedures collectively accounted for less than 2.0% of treatments. Table 4 shows the frequency of the different dental treatments during the sessions under anaesthesia.

**Table 4:** Intervention of dental treatment

	Absolute frequency [*n*]	Relative frequency [%]
Total number of patients	154	100.0
Type of dental intervention		
Restorative therapy	52	33.8
Tooth extraction	57	37.1
Combination of restorative therapy & tooth extraction	42	27.2
Osteotomy surgery	3	1.9

### Anaesthesiological and general peri- and postoperative parameters

Dental treatment under GA was performed in 143 patients (92.86%), and AS was used in 11 cases (4.55%). The majority of patients undergoing GA were intubated orotracheally, and more than two-thirds (66.9%) of the total patient population received antiemetics (Table 3). Inpatient care was mainly provided by the neurological paediatric unit (35.1%) and the general paediatric unit (28.6%). 22.1% of the patients were treated exclusively in outpatient clinics and went at home the same day after receiving postoperative care. Complications or side effects of any kind occurred in less than 3.0% of cases (Table 4). None of the patients required pre- or postoperative intensive care. No patient required resuscitation. Two patients required reintubation. Four patients (3 GA/1 AS) vomited in the hospital following the procedure and 3 patients (2 GA/1 AS) showed allergic reactions (Table 5), one showing rash and the others showing flushing.

**Table 5:** Absolute and relative frequency for peri- and postoperative setting

	Absolute frequency [*n*]	Relative frequency [%]
Total number of patients	154	100.0
Type of intubation		
No intubation	11	7.1
Orotracheal	142	92.2
Nasotracheal	1	0.6
Administration of antiemetics		
yes	103	66.9
No	51	33.1
Inpatient care		
Day clinic	34	22.1
General paediatric unit	44	28.6
Neurological paediatric unit	54	35.1
Paediatric cardiac unit	11	7.1
Oncological paediatric unit	2	1.3
Combination of general paediatrics and paediatric cardiac unit	1	0.6
Combination of neurological paediatric and paediatric cardiac unit	2	1.3
Combination of general paediatrics and oncological paediatric unit	1	0.6
Intensive care unit	0	0.0
Other	4	2.6
Complications/side effects		
Resuscitation	0	0.0
Allergic reaction	3	1.9
Reintubation	2	1.3
Unplanned admission to intensive care unit	0	0.0
Nausea/vomiting	4	2.6

## Discussion

In the present study, a highly specific patient population from a period of three years was examined enabling a comparison with other studies in the literature (Campbell et al. 2018; Hung et al. 2003).

Data exhibited genetic syndromes in a high proportion, followed from the children with cardiac findings and metabolic disease. The groupings of the disease forms presented in Delfiner et al. largely coincide with the present categorisation (Delfiner et al. 2017). In contrast to our study, Delfiner et al. could not identify a predominant form of disease; all forms of disease were relatively evenly distributed (Delfiner et al. 2017). The majority of children with genetic syndromes and cardiological findings may be explained by the close collaboration with paediatric cardiology and paediatric dentistry in the Paediatric Heart Center. It is reasonable to assume that parents have their children treated in an institution that has detailed knowledge of the sometimes complex history and treatment of their children and has been consistently involved in this treatment on a multidisciplinary basis (Sprong et al. 2024). Moreover, dental rehabilitation is also routinely carried out at the Children’s Heart Centre.

Regarding dental procedures, restorative therapy and its combination with surgical procedures have accounted for a large proportion of the treatments (summarised 61% of total treatments). This agrees with the results of Mallineni et al. (2016), where restorative therapy was also the most prevalent dental procedure (Mallineni and Yiu 2016). Rajavaara et al. report in their retrospective analysis of paediatric GA cases that the number of fillings in healthy patients is smaller compared to those in patients with SHCN, but the number of pulpotomies is larger (Rajavaara et al. 2017). However, it is important to note that endodontic measures such as vital amputations are not carried out for focal restorations in children with congenital heart disease or children with tumour diseases, so that more extractions may have taken place here. This fact is also in accordance with Tewfik et al. showing more extractions vs restaurative care in SHCN patients (Tewfik et al. 2023). Ciftci and Yazicioglu (2020) also reported a higher number of extracted teeth in children with SHCN treated under GA compared to healthy children (Ciftci and Yazicioglu 2020). Moreover, we found significant differences between the type of general anaesthesia and the dmf-t value for the primary dentition. The second null hypothesis can therefore be rejected. This can be explained by the fact that GA is often used for large dental findings and AS is generally used to treat lower dental findings. The term AS describes the utilisation of various analgesic and sedative medications to facilitate medical examinations, interventions, or surgical procedures in spontaneously breathing patients. Following the establishment of intravenous access, analgesics and sedatives can be administered either continuously or intermittently. The primary objectives are maintenance of effective spontaneous respiration, haemodynamic stability, and simultaneous tolerance of the intervention. In our centre, the pharmacological agents employed in the described patient cohort include benzodiazepines, NMDA receptor antagonists, disoprivan, α2-adrenoreceptor agonists, and opioids. The selection of specific agents is individualised based on patient-related factors and drug-specific characteristics. Patient monitoring in this context encompasses ECG, pulse oximetry, non-invasive blood pressure measurement, and capnography. Based on our own experience, anaesthesia procedures that guarantee a secure airway, such as GA, are chosen for therapies involving water cooling.

Regarding dmf-t/DMF-T value, the first null hypothesis could be rejected too, due to different dmf-t/DMF-T value for every general disease with highest value for the primary dentition in patients with tumour disease vs highest for genetic syndrome patients in permanent dentition. One reason for this could be that the children with tumour diseases have to be rehabilitated due to urgent focal rehabilitation with significant findings. The older syndrome children are only treated under GA if compliance does not permit dental treatment chairside.

In addition, there was a significant difference in the dmf-t value for the deciduous teeth with regard to the type of anaesthesia. Patients treated under GA had a significantly higher value than AS patients (see Fig. 2). This can be explained by the fact that AS is generally only chosen for the rehabilitation of minor oral findings. A minor oral finding corresponds to a lower dmf-t value.

The age of dental treated patients under GA was higher than in other studies, such as in the German study by Takriti et al. (2019), in which the average age of hospital patients was 4.65 years (Takriti et al. 2019). This higher age in patients with higher ASA status, i.e. with increasingly severe general disease, is in accordance with the literature showing oral rehabilitation at an older age than children without general diseases (Delfiner et al. 2017; Escanilla‐Casal et al. 2016). Besides this, the relatively high age of the patients is also caused by waiting times ranging from months to a year due to the high demand for treatment under general anaesthesia (Schulz-Weidner et al. 2022).

The gender distribution with a slightly higher number of males (81 males, 73 females) treated under GA is confirmed by other comparable studies (Takriti et al. 2019).

With regard to the perioperative complications that occurred in the observation of this vulnerable patient group, there were no cases which needed a resuscitation. There is very little data available in the literature on resuscitation measures and deaths during dental procedures under general anaesthesia. However, it is reported that deaths occurred mainly outside the hospital setting and when the general anaesthesia was administered by non-anaesthesiologic staff (Lee et al. 2013). A controlled environment, highly trained and specialised practitioners and precise, predetermined therapy- and care-goals may have influenced these positive outcomes (Ramsay and Rappaport 2011; Safetots 2005). However, since the group studied was significantly sicker than the general population, the low complication rate should be emphasised.

Furthermore, none of the patients underwent an unplanned admission to intensive care unit. It is suggested that dental procedures under GA lead less frequently to unplanned inpatient stays than other surgical procedures (Lucy et al. 2017).

It is further described that unplanned inpatient admissions after dental procedures under anaesthesia are associated with vomiting for children of ASA I-IV (Lucy et al. 2017). In this study, postoperative nausea and vomiting occurred only in four cases (2.6%) during the surgical stay on the unit. Other studies report higher relative numbers, such as 20–25%, of PONV after dental treatment under general anaesthesia for healthy patients (Apipan et al. 2016; Cantekin et al. 2014) and 33.2% to 82% in paediatric surgery (Eberhart et al. 2004). In this study, the prevention of PONV was approached with a drug-based approach by the administration of ondansetron as antiemetic substrate in most of the cases underlining the good effectiveness. According to the literature, PONV is decreasing when propofol is used for GA (Yumura et al. 2011), which was used for total intravenous sedation in our patient group. Moreover, during surgical treatments with bleeding, particular attention was paid to good suction and haemostasis to avoid nausea caused by swallowing blood. Risk factors for the occurrence of PONV can be subdivided into surgical, anaesthetic and patient factors. The main identified risk factors for PONV include the intraoperative administration of opioids during the recovery room stay and at the ward, the intraoperative use of non-opioids and the specific type of surgical procedure (Messerer et al. 2023). The emetogenic effect of opioids was minimised in the group studied here by the oral application of local anaesthetics by dentists. The influence of the dental intervention appears to be secondary to patient and anaesthesiological factors (Apipan et al. 2016).

The literature also reports about nasal bleeding as a postoperative complication after general anaesthesia (Cantekin et al. 2014). In the present study, the patients were mainly intubated orally, which per se prevents the occurrence of nasal bleeding. However, oral intubation can also be a disruptive factor during interventions in the oral cavity. For example, the extent to which this circumstance influenced the duration of surgery was not recorded in this study.

However, reintubation was necessary in two cases that were intubated orally. The need for reintubation may have been due to excessive manipulation of the tube during oral intubation and time-consuming reintubation work due to limited space in the oral cavity. This is a disadvantage of oral intubation during procedures in the oral cavity and should be considered and monitored by both the dentist and the anaesthetist.

Allergic reactions in temporal association with the anaesthesia were recorded in three patients. One of them showed skin changes and the two a flush reaction at the beginning of the anaesthesia procedure. It is described that flush reactions are often related to the induction of the anaesthesia and reagents, such as muscle relaxants (Olday et al. 2003). In these cases, it was not recorded whether the triggering substance was identified postoperatively in the affected patients. According to Dippenaar and Naidoo (2015), both of the phenomena that occurred are attributable to the lowest grade of allergic reactions and anaphylaxis during anaesthetic (Dippenaar and Naidoo 2015). Due to the small number of complications, we were unable to detect any differences in the various disease groups, which is why the third null hypothesis can be confirmed.

The results of this study show that very few complications occur with regard to the anaesthesia, which indicates a safe procedure for dental rehabilitations in this vulnerable group of SHCN patients underlining the need of good collaboration of anaesthetists and dentists and risk mitigation protocols to maintain the often rare possibility of being able to safely treat children and adolescents with severe general diseases under G with regard to oral findings.

Although a dental treatment under GA can serve as a full-mouth rehabilitation within one session, it should be born in mind that most of the patients are at a high risk level to develop new caries lesions (Mathew et al. 2024). Patients with a history of ECC are generally at a higher risk of caries in the permanent teeth (EzEldeen et al. 2015). Showing that children with special healthcare needs are a group with a high caries risk highlights the need for preventive dentistry in these patients. Therefore, it is important to keep the patients adhered to a strict recall and prophylaxis programme to avoid the need for further treatments under GA, especially in these high-risk SHCN patient groups.

The main limitation of our study is that it was conducted at a single centre and only included children with SHCN. Moreover, no age groups were compared, which might have changed the results regarding the type of intubation. In addition, there was no calibration of the investigators due to the retrospective nature of the data collection. Therefore, prospective study designs are recommended for further investigation, but as the practitioners had the same training in their undergraduate studies and postgraduate training in the department, this factor is likely to be negligible. Confirmation of our results with a larger number of children enrolled and longer follow-up would be desirable.

## Conclusions

Data exhibited high caries experience in SHCN patients regardless of general disease, with a need for dental treatment. Due to the extensive restoration findings, most dental restorations were carried out under general anaesthesia. Regardless of disease, there was a low rate of complications underlining the high clinical relevance of appropriate dental care under GA for this vulnerable group of patients.

## Data Availability

The datasets in this article are available from the corresponding author upon reasonable request.

## Abbreviations

ASA: American Society of Anaesthesiologists
AS: Analgosedation
dmf-t/DMFT: Sum of the decayed(d/D), missing (m/M) and filled (f/F) teeth
ESPA: European Society for Paediatric Anaesthesiology
GA: General anaesthesia
IQR: Interquartile range
PONV: Postoperative nausea and vomiting
SD: Standard deviation
Safetots: Safe anaesthesia for every tot
SHCN: Special healthcare needs
